# Comparison of Hemodynamic and Recovery Profile Between Segmental Thoracic Spinal and General Anesthesia in Upper Abdominal and Breast Surgeries: A Systematic Review and Meta-Analysis

**DOI:** 10.7759/cureus.68792

**Published:** 2024-09-06

**Authors:** Habib Md R Karim, Imran A Khan, Arshad Ayub, Ghazal Ahmed

**Affiliations:** 1 Anesthesiology, Critical Care, and Pain Medicine, All India Institute of Medical Sciences, Guwahati, Guwahati, IND; 2 Community Medicine, Baba Raghav Das Medical College, Gorakhpur, IND; 3 Community and Family Medicine, All India Institute of Medical Sciences, Deoghar, Deoghar, IND; 4 Dermatology, Venereology, and Leprosy, All India Institute of Medical Sciences, Deoghar, Deoghar, IND

**Keywords:** adult patient satisfaction, cholecystectomy laparoscopic, ethical and legal principles in medical practice, general anaesthesia, general surgery breast cancer, intraoperative bradycardia, intraoperative hypotension, patient recovery, postoperative nausea vomiting, thoracic segmental spinal anesthesia

## Abstract

Segmental thoracic spinal anesthesia (STSA) has been described primarily as case reports for performing upper abdominal and thoracic surgeries in significant respiratory comorbid patients. A few comparative studies have recently evaluated the technique as an advantageous alternative to general anesthesia (GA). However, there is no systematic evaluation and comparison of the techniques. The present systematic review evaluated the hemodynamic, comfort, and satisfaction of patients undergoing abdominal and thoracic surgeries under STSA and GA. PubMed, CENTRAL, Google Scholar Advanced, and citation tracking were performed to find suitable articles that compared STSA and GA. The primary objective-related data were hypotension and bradycardia. The secondary objective-related data in the context of postoperative nausea vomiting (PONV), pain, rescue analgesics, sedation requirement, satisfaction, and comfort were assessed. Meta-analysis was performed for dichotomous data on hypotension, bradycardia, and PONV; odds ratio (OR) and 95% confidence interval (CI) were reported. Data of 394 patients from six studies were evaluated. Patients undergoing upper abdominal and breast surgeries under STSA had significantly higher odds of hypotension (Fixed-Effect Model OR 12.23, 95% CI 2.81-53.28; I2 =0%, and the Random Effects Model OR 12.01, 95% CI 2.75-52.52; I2 =0%) and bradycardia (Fixed-Effect Model OR 10.95, 95% CI 2.94-40.74, I2 =0%, and the Random Effects Model OR 9.97, 95% CI 2.61-38.08; I2 =0%) but lower odds of PONV (Fixed-Effect Model OR 0.24, 95% CI 0.13-0.43; I2 =0%, and the Random Effects Model OR 0.24, 95% CI 0.13-0.45; I2 =0%). Most of the patients undergoing STSA were given intravenous sedation to overcome anxiety and discomfort. Overall, patient satisfaction was on par with GA. However, few surgeons were unenthusiastic about the technique while performing axillary clearances due to bothering twitches from cautery. STSA led to early post-anesthesia care unit (PACU) discharge and provided better pain control, lowering the need for rescue analgesics and opioid consumption in the first 24-hour postoperative period. STSA is associated with very high odds of hypotension and bradycardia as compared to GA. On the other hand, STSA demonstrated superior pain control, reduced opioid requirements, shorter PACU stays, and significantly reduced risk of PONV. Nevertheless, STSA patients mostly require sedation to make the patient comfortable.

## Introduction and background

Segmental thoracic spinal anesthesia (STSA) is gaining interest [1,2,3]. Although the technique is not unknown and the performance of laparoscopic cholecystectomy was reported nearly two decades ago [4], the procedure was mainly looked upon as an alternative to general anesthesia (GA), where GA was considered at higher risk of morbidity and mortality due to patients' comorbid conditions [5]. However, the outlook is being challenged, and a few publications have given us a second thought [5,6]. The excellent point about STSA is that it is a regional anesthesia (RA) technique. Given the established superiority of RA techniques over GA with better recovery time, respiratory dynamics, and postoperative analgesia, STSA is recently gaining popularity for upper abdominal and breast surgeries [2,4,6].

The STSA technique avoids morbidity related to tracheal intubation, neuromuscular blockade, and administration of positive pressure ventilation, which positively impact the patient outcome, especially in frail and comorbid patients with respiratory compromises [5]. Aljuba YM et al. have reported a case of emergency open cholecystectomy in a past heavy smoker, morbidly obese elderly chronic obstructive pulmonary diseases (COPD) patient requiring domiciliary oxygen therapy who had grossly limited functional activity (New York Heart Association functional status class III), pulmonary artery hypertension (55 mmHg), and frequent premature atrial contractions under combined epidural spinal anesthesia where the spinal anesthesia component was STSA [7]. The anesthesia was successfully conducted, which could avoid using paralytic agents and positive pressure ventilation, considered risks for or exacerbation of respiratory failure. Another good aspect is the potential use of the technique to provide opioid-free or opioid-sparing anesthesia [2,6]. Perioperative opioids as analgesia and sedation are time-tested and fail-proof drugs but have been looked upon recently as an avoidable armamentarium associated with potential adverse outcomes like nausea, vomiting, postoperative respiratory depression, and even possible addiction [8].

Nevertheless, using the technique for routine upper-abdominal or thoracic surgeries remains controversial and debated. A comprehensive and systematic review is also needed to analyze the impact of anesthesia techniques on hemodynamics and other perioperative morbidities. Therefore, we aimed to conduct this systematic literature review to assess the technique's safety regarding hemodynamic outcomes primarily as an alternative to GA for upper abdominal and breast surgeries. We also planned to review and analyze the impact of STSA on pain, analgesia, recovery, and nausea and vomiting as secondary outcomes.

## Review

Methods

The non-registered systematic review and meta-analysis followed the Preferred Reporting Items for Systematic Reviews and Meta-Analysis (PRISMA) statement guidelines.

Search Strategy and Data Collection

Two authors (HK and IK) independently and systematically performed literature searches on PubMed, Cochrane Central Register of Controlled Trials, and Google Scholar until May 2024. Search terms included the keywords and the corresponding index words for segmental thoracic spinal anesthesia in the advanced search feature with different filters, as shown below in Table 1.

**Table 1:** Search summary with database name and index word combinations.

Database/Engine	Index word/Combinations	No. of articles
PubMed Advanced	("segmental thoracic spinal"[All Fields]) AND ("general anesthesia"[Title])	3 articles
PubMed Advanced	("segmental thoracic spinal"[All Fields]) AND ("general anaesthesia"[Title])	Nil
PubMed Advanced	(("segmental thoracic spinal"[Title/Abstract]) OR ("segmental thoracic spinal anesthesia"[Title/Abstract]))	15 articles
PubMed Advanced	((("segmental thoracic spinal"[Title/Abstract]) OR ("segmental thoracic spinal anesthesia"[Title/Abstract])) OR ("thoracic spinal anaesthesia"[Title/Abstract])) OR ("thoracic spinal anesthesia"[Title/Abstract])	33 articles
Cochrane (CENTRAL)	segmental thoracic spinal	119 records
Google Scholar Advanced	segmental thoracic spinal (with all the words) in Title, Filter: include citation not selected	80 results
Google Scholar Advanced	segmental thoracic spinal (with all the words) in Title, Filter: include citation selected	106 results

Further, the reference lists of the studies from PubMed and CENTRAL, whose full-text retrieval was done, were also screened (citation tracking) for additional relevant studies.

Inclusion and Exclusion Criteria

Eligible studies were any article presenting primary data as a comparative, observational, or non-randomized and randomized controlled trial that fulfills our key concepts under population, intervention, comparator, outcomes, and study (PICOS), as summarized in Table 2.

**Table 2:** PICOS of the present review. PICOS: Population, Intervention, Comparison, Outcomes, and Study; STSA: segmental thoracic spinal anesthesia

Particulars	Description
P: Populations	Patients undergoing abdominal and thoracic region surgeries
I: Intervention	Segmental thoracic spinal anesthesia
C: Comparator	General anesthesia
O: Outcome	Primary: Hemodynamic (hypotension and bradycardia); Secondary: Need for analgosedation in the STSA group; Secondary: Patient and surgeon satisfaction
S: Studies	Original article with comparison, both randomized / non-randomized and observational.

The literature was searched in English, excluding incomplete information, conference abstracts, reviews, feasibility studies, and dissertations. We also excluded thoracic spinal anesthesia performed along with other types of blocks or epidurals. While we did not strictly adhere to our inclusion outcome to report predefined outcomes, reporting hemodynamics regarding the incidence of hypotension and bradycardia was our primary target.

Data Extraction

Two researchers (Author IK and GA) independently audited the literature and extracted data according to the inclusion and exclusion criteria. A manual screening method was employed to remove identical literature; titles and abstracts were evaluated for initial screening, and literature that did not fit our review objective was excluded. Full-text extraction was done (by Authors HK and IK) and assessed for final inclusion based on the methodology employed. We emailed the authors for the full text and missing data as required.

Data extraction included the study's first author, the year of publication, the number of subjects, sex, interventions, and outcome as per our PICOS. Where there was disagreement, HK resolved the disagreement and made decisions after a joint discussion.

Statistical Analysis

The meta-analysis statistics were performed using R version 4.3.2, and the OR with a 95% confidence interval (CI) was calculated along with the weightage of the studies. The heterogeneity among trials was assessed using Higgins’ and Thompson's I2. The OR for each study was plotted as a square with a horizontal line extending from it, representing the 95% CI. The squares represent the effect size, and the size of each square reflects the weight assigned to that study in the analysis. The percentage weight assigned to each study is shown for the common and random effects models. A p-value < 0.05 was considered significant.

The risk of bias was independently assessed by two authors (HK and AA); for RCTs, the risk of bias was assessed using the risk of bias-2, and for non-randomized observational studies, the risk of bias was assessed using the risk of bias in non-randomized studies of interventions (ROBINS-I) tool. The visualizations were created using risk-of-bias VISualization (robvis) [9].

Results

**Figure 1:**
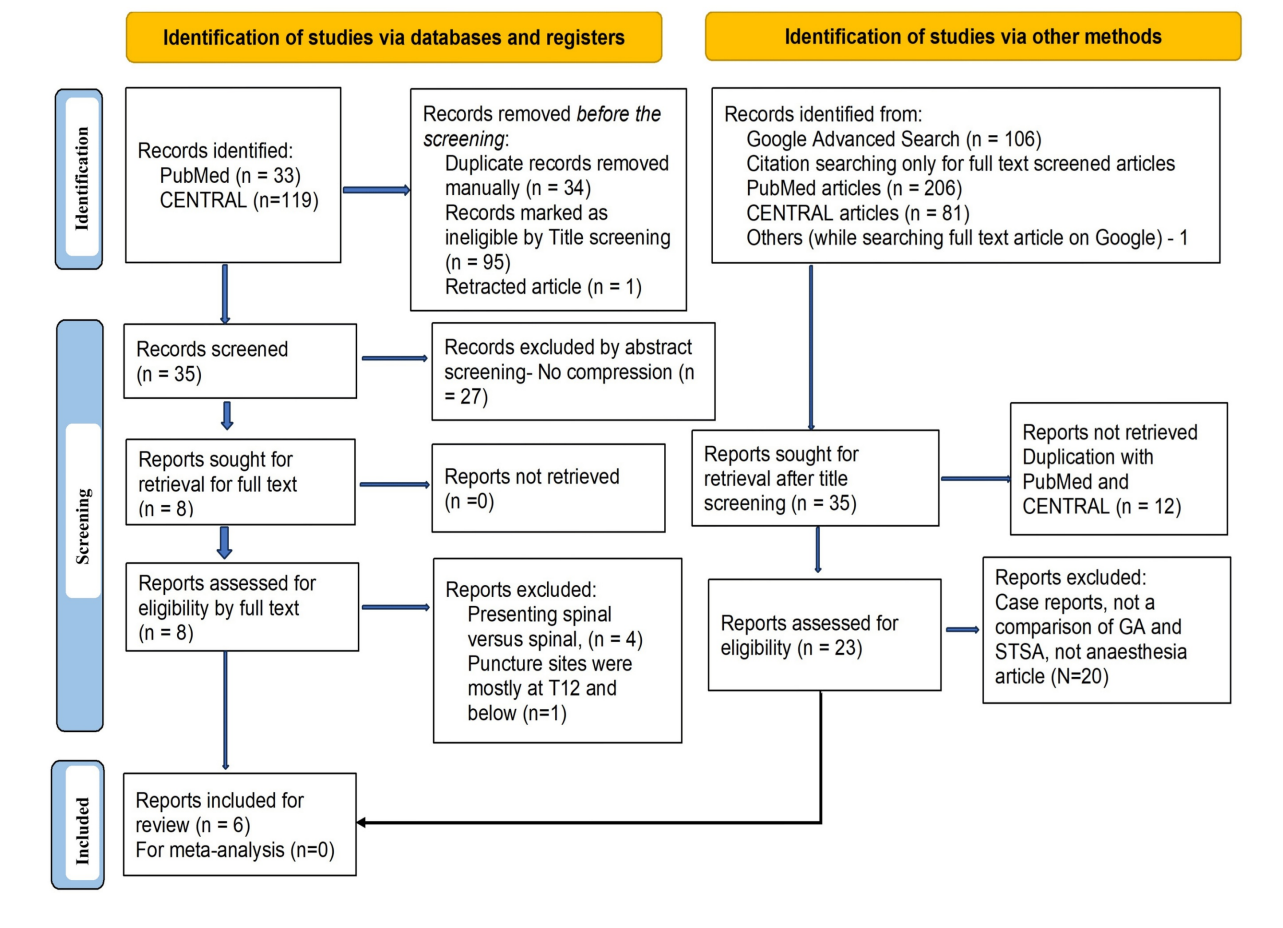
PRISMA 2020 flow chart. PRISMA: Preferred Reporting Items for Systematic Reviews and Meta-Analysis; GA: general anesthesia; STSA: segmental thoracic spinal anesthesia

A PubMed and CENTRAL search using predefined Boolean index words in different combinations yielded a maximum of 33 and 119 articles, respectively. A Google Scholar advanced search revealed 106 results. Further, citation tracking of 12 papers from PubMed and CENTRAL, whose full texts were screened, provided 287 references. Finally, six articles were included for the present review after the title, abstract, and full-text screening and applying the PICOS (PRISMA flow diagram, Figure 1).

The table presents data related to the authors, years of publication, number of participants, level of spinal arachnoid puncture, and primary and secondary objectives of the included six studies (Table 3) [10-15].

**Table 3:** Summary of the included studies. GA: general anesthesia, PONV: postoperative nausea vomiting, RCT: randomized controlled trial, STSA: segmental thoracic spinal anesthesia; PACU: post-anesthesia care unit

Sl. No.	Authors (Year)/Country	Type of study	ASA-PS, No. (Sex/ Age)	Spinal level	Spinal medications (total volume)	Type of surgery	Hemodynamics	Comfort, sedation, satisfaction, hospital stay
1	Elakany MH, et al. (2013) / Egypt [10]	RCT study (STSA vs. GA)	I-III 40 F / 20-65 years.	T5-6	Bupivacaine (I) 5 mg + 20 μg fentanyl	U/L mastectomy with axillary dissection	Hypotension and bradycardia (15%) were more frequent in the segmental thoracic spinal group than in GA (0.0%). In contrast, hypertension and tachycardia were seen in 20% and 30%, respectively, in the GA group and none in the STSA group.	PONV and patient satisfaction were statistically indifferent. PACU discharge times were shorter in the STSA group.
2	Elakany MH (2014) / Egypt [11]	Comparative study (STSA vs GA)	II-III 60 (37 M, 23 F/20-70 years)	T9-10	Bupivacaine 10 mg + 20 ug fentanyl	Abdominal carcinoma resections	6 (20%) suffered hypotension, and 6 (20%) had bradycardias in STSA versus none in the GA. No data on hypertension and tachycardia.	Six (20%) had anxiety, and three (10%) had PONV in STSA versus none in GA. Two (6.67%) had paraesthesia in STSA. The length of stay in the recovery room and the hospital was shorter in STSA, and better analgesia and patient satisfaction were noted.
3	Haq HU et al. (2022) / India [12]	RCT (GA and STSA)	I-II 90 (29M and 61 F/18-60 years)	T10-11	5 mg Bupivacaine (I) 0.5%, + 25 mcg fentanyl	Laparoscopic cholecystectomy	2 (4.4%) had bradycardia, and 5 (11.1%) had hypotension in STSA versus none in the GA.	PONV was higher at 26.7% in GA than 11.1% in STSA, but the difference was statistically indifferent. Intraoperative right shoulder pain/discomfort in the STSA was noted. STSA showed the superiority for better postoperative pain control as compared to GA.
4	Paliwal N et al. (2022) / India [13]	RCT (GA vs. STSA) Non-blinded study.	I-II 56 (Female)	T5-6	5 mg of Levobupivacaine (I) 0.5% + 20µg of fentanyl	Breast surgery	Eight (28.5%) had bradycardia in STSA. No hypotension was noted.	Three (10.71%) patients experienced paresthesia during spinal puncture without sequelae. Surgeon and patient satisfaction were better in STSA. Significantly lesser opioid consumption in the postoperative period in STSA. PONV was 25% in GA as compared to 3.75% in STSA. The length of stay in the recovery room and the hospital was shorter in group TS than in group GA.
5	Gupta N et al. (2024) / India [14]	Comparative study (STSA vs. GA)	II-III 58 (F 60/ 18-60 years)	T4-5	7.5 mg levobupivacaine (H) 0.5% + 15 mcg fentanyl	U/L mastectomy with axillary clearance	There are no direct data. We emailed the corresponding author, but we did not receive a response until the manuscript was finalized.	Two (6.67%) STSA cases converted to GA. The entire STSA provided intravenous sedation during axillary clearance. Surgeons were not enthusiastic about STSA as twitch responses to cautery were bothering. Induction time was higher in the STSA than in GA. PACU discharge readiness was better in STSA, but there were higher lengths of hospital stay than in GA.
6	Goel L et al. (2022) / India [15]	RCT (GA versus STSA)	I-II 90 (45 M and 45 F / 18-60 years)	T10-T11	5 mg of Bupivacaine (I) 0.5% + 25µg of fentanyl	Laparoscopic cholecystectomy	Two (4.4%) bradycardia and five (11.1%) hypotension in the STSA group versus none in the GA group. Group STSA shows less tachycardia (exact value not reported).	PONV was higher at 26.7% in GA than 11.1% in STSA, but the difference was statistically indifferent. Intraoperative shoulder pain and discomfort were noted in the STSA group. Discharge time and satisfaction data were not provided; only mentioned in the conclusion was shorter and better in the STSA. There were fewer procedure-related costs and hospital stays in STSA.

These six articles included 394 patients of both genders. Two of these articles were comparative, non-randomized studies, and four were RCTs. The study by Haq HU et al. [12] and Goel L et al. [13] appears to be similar in context to the patients recruited, surgeries performed, settings, and time duration. Even the results, interpretation, and content of the articles are identical. However, they were not excluded because they were published in different journals and do not come under citation duplication. Nevertheless, publication misconduct cannot be ruled out. The most common surgery conducted under thoracic segmental spinal was laparoscopic cholecystectomy (180, 45.45%), followed by breast surgery (156, 39.39%), and the rest were abdominal cancer surgeries.

Variations in the level of spinal arachnoid puncture and drugs used were noted for STSA. Three studies used high (T4-6) thoracic interspace. Thoracic interspaces T4-6 in most breast surgeries, T9-10 in laparoscopic cholecystectomy, and T10-11 in abdominal surgeries. Four (66.67%) studies used bupivacaine, and two used levobupivacaine. The dose for bupivacaine ranged from 5 to 10 mg, while for levobupivacaine, the dose ranged between 5 and 7.5 mg. The entire study also used fentanyl as an adjuvant at a dose ranging from 15 to 25 mcg. Plain/isobaric drugs were mainly used for STSA.

Regarding the GA technique, the entire study used balanced anesthesia, and the induction and maintenance were similar. However, none of the studies indicated whether the minimum alveolar concentration monitoring was adjusted for age and targeted to a specified value [16,17]. The entire research used neostigmine and glycopyrrolate for reversal. The studies also did not objectively report the risk of postoperative nausea and vomiting and practice details for prevention. Further, the entire GA conducted needed a proper description of the multimodal analgesia techniques.

**Figure 2:**
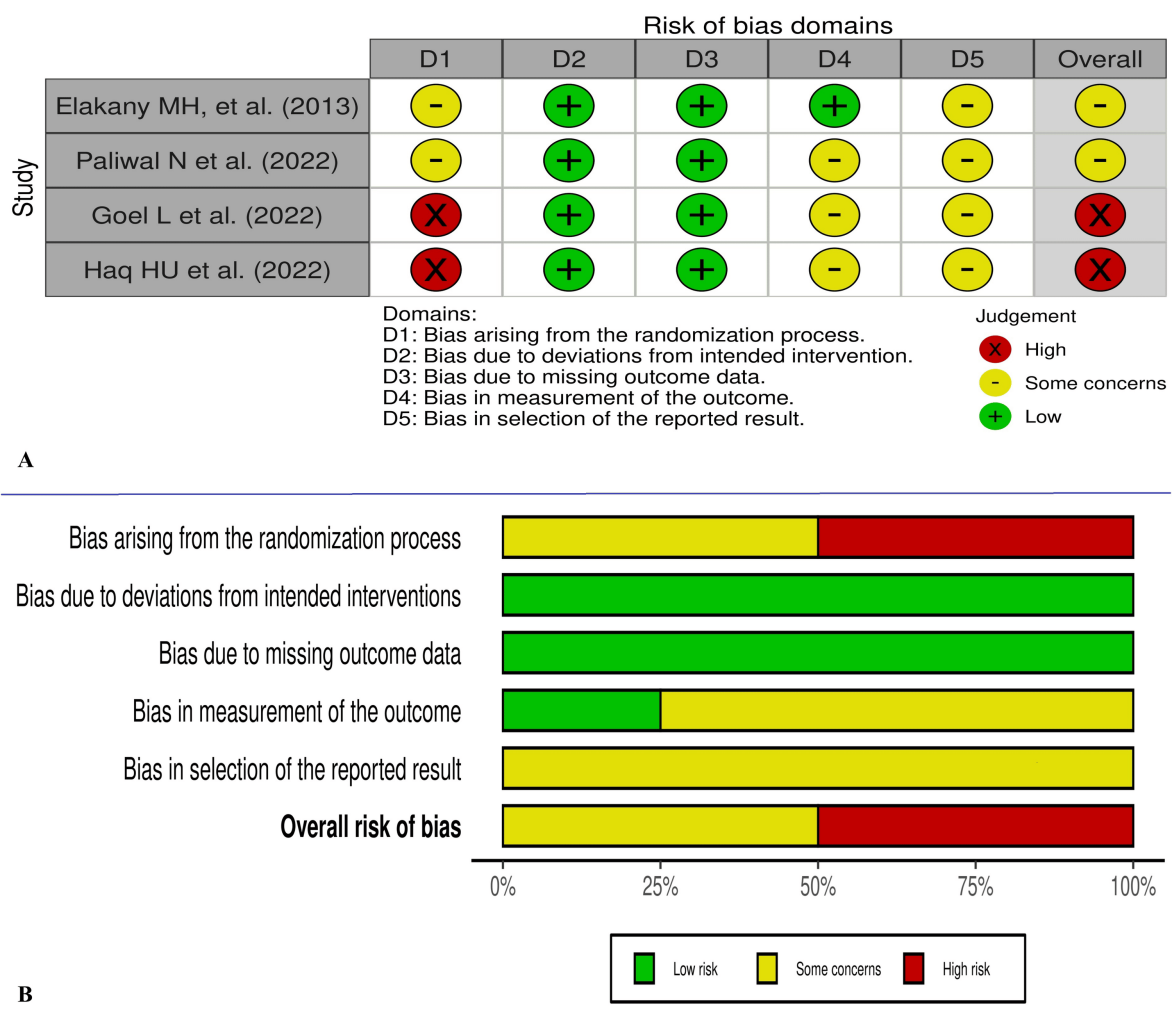
Traffic light (A) and summary visualization graph (B) for risk of bias as per RoB-2 for the RCTs included. RoB-2: risk of Bias assessment tool version 2; RCTs: randomized controlled trials The images were created using risk-of-bias VISualization (robvis) [9]. [10,12,13,15]

**Figure 3:**
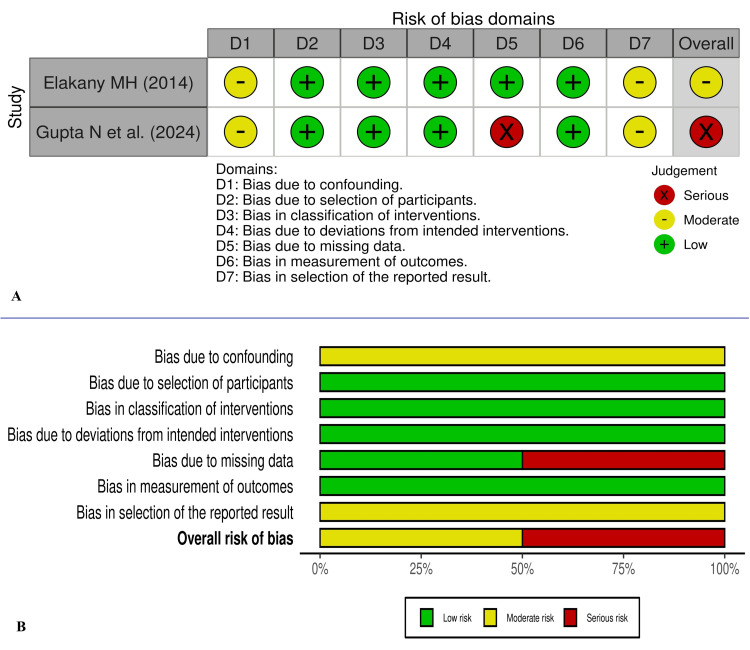
Traffic light (A) and summary visualization graph (B) for risk of bias as per ROBINS-I for the non-randomised studies included. ROBINS: risk of bias In non-randomized studies of interventions The images were created using risk-of-bias VISualization (robvis) [9]. [11,14]

The study by Haq HU et al. [12] and Goel L et al. [15] appears to have major concerns about randomization, and Paliwal et al.’s study [13] is non-blinded. Most of the included studies also have other concerns about biases. The RoB-2 and ROBINS-I visualization images for risk of biases are presented in Figures 2, 3, respectively.

Five of the six studies reported hypotension and bradycardia data, which are presented in the table. Gupta et al.’s study did not directly report the number of patients with hypotension and bradycardia [14]. We emailed the corresponding author for the data but did not get a response until the manuscript was written. Observing the table and graphs of the hemodynamics showed that the STSA group had fewer hemodynamic parameter variations than Group GA and showed less tachycardia and hypertension.

**Figure 4:**
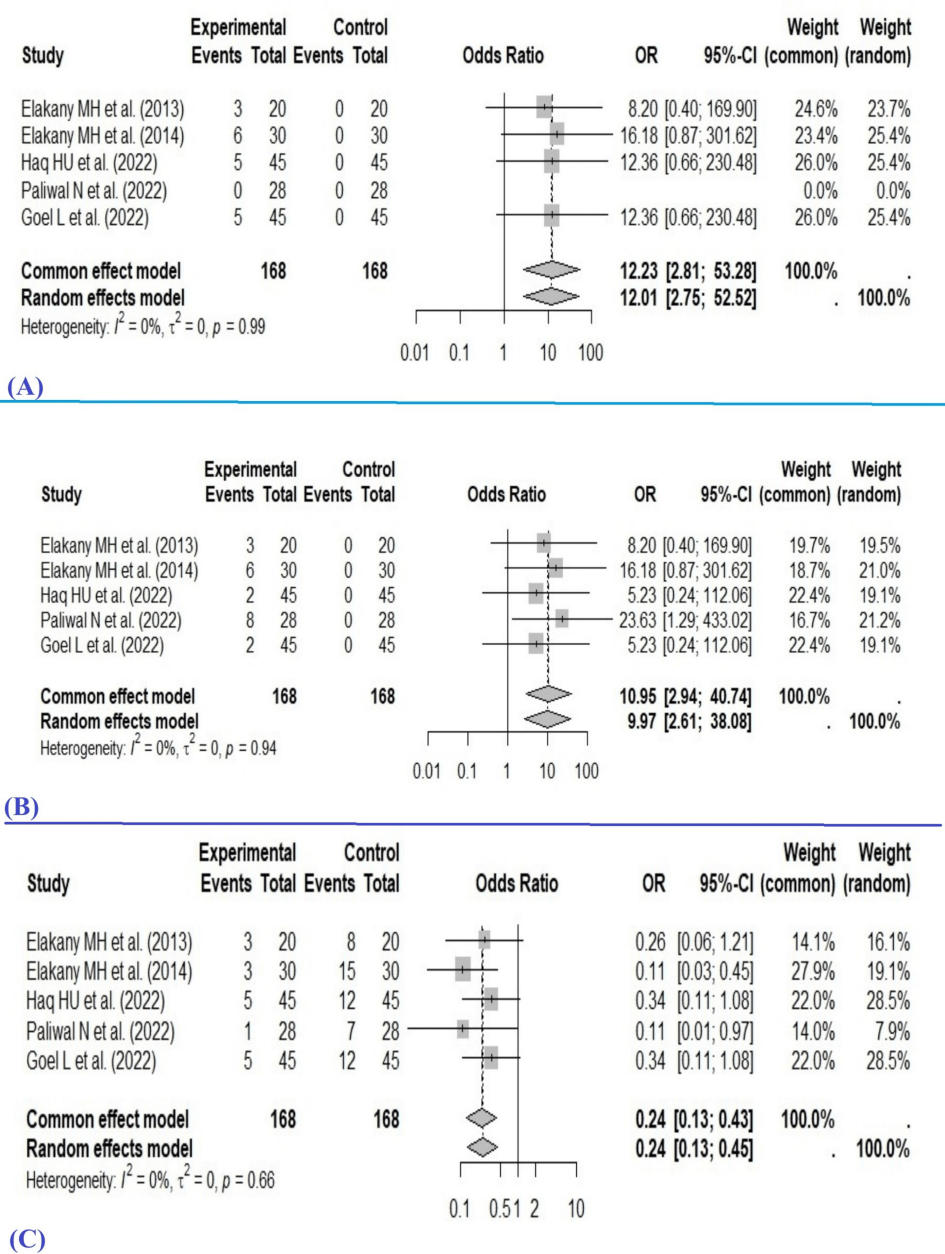
Forest plot for (A) hypotension, (B) bradycardia, and (C) PONV. PONV: postoperative nausea and vomiting [10,11,12,13,15]

The Common Effect Model (Fixed-Effect Model) found the pooled OR as 12.23 (2.81-53.28), and the Random Effects Model found the pooled OR as 12.01 (2.75-52.52) for the hypotension in the STSA group when compared with GA. On the other hand, the Common Effect Model (Fixed-Effect Model) showed the pooled OR as 10.95 (2.94-40.74) for the development of bradycardia with STSA, and the Random Effects Model showed the pooled OR as 9.97 (2.61-38.08). The pooled OR from both the common effect and random effects models indicates that the intervention in the experimental (i.e., STSA) group has significantly higher odds of developing hypotension and bradycardia than the control (i.e., GA). Heterogeneity testing for hypotension across the included studies showed I² =0%, τ² =0, p =0.99. The statistical values for bradycardia are I² =0%, τ² = 0, p =0.94, suggesting no observed heterogeneity among the studies (Figure 4).

The Common Effect Model (Fixed-Effect Model) found the pooled OR as 0.24 (0.13-0.43), and the Random Effects Model found the pooled OR as 0.24 (0.13-0.45) for the PONV in the STSA group when compared with GA. Heterogeneity testing for PONV across the included studies showed I² =0%, τ² =0, p =0.66.

There were conflicting results. While Paliwal et al. [13] found that STSA led to better patient and surgeon satisfaction, and Elkany MH [11] found better patient satisfaction in breast and abdominal surgeries, the study by Gupta et al. [14] showed that cautery-induced twitches during axillary resection annoyed and troubled the surgeons. Goel et al.’s study reported intraoperative right shoulder pain/discomfort in the STSA [15]. Anxiety and discomfort were concerns in the STSA, and sedatives were used to control them. All participants of the STSA in Paliwal et al.’s study and Gupta et al.’s study required intravenous sedatives [13,14]. Most studies found that STSA significantly improved pain control and the requirement for rescue analgesics compared to GA. Paliwal et al. also found substantially lower opioid consumption in the postoperative period [13]. The STSA group had a shorter post-anesthesia care unit (PACU) study [10,11]. However, it did not lead to a uniform, shorter hospital stay [14]. The length of stay in the recovery room was shorter in STSA, while the length of hospital stay showed variable results [10,13,14]. The total anesthetic cost was lower in the STSA than in GA [12].

Discussion

The present analysis indicates that STSA might be used for upper abdominal and breast surgeries in the American Society of Anesthesiologists Physical Status (ASA-PS) I-II and probably even in ASA-PS III patients. Although STSA use is often argued for patients with cardi-pulmonary compromise [3], none of the studies evaluated in-depth respiratory parameters in the STSA. Hypotension and bradycardia were significantly higher in the STSA group. Nevertheless, the incidence of PONV was significantly lower in the STSA group. Further, the STSA provided PACU discharge readiness early, had better pain control, and required fewer rescue analgesics and opioids in the immediate post-24-hour period.

Multimodal analgesia is the analgesia technique in the current minimum standard expected for intraoperative and postoperative pain management, which has the advantages of opioid-sparing and enhanced recovery [18]. Regional analgesia techniques play a crucial role as a component whenever possible while designing multimodal analgesia [19,20]. Therefore, the benefits obtained for acute pain control and related rescue analgesia for the STSA technique can be well explained as the residual effect and prolongation of the effects of the fentanyl adjuvants aided in providing ideal multimodal analgesia in the intraoperative and postoperative period. On the contrary, GA group patients were not offered the benefit of regional analgesia, which is the usual practice nowadays [18,19,20]. Such multimodal analgesia can help recover patients undergoing upper-abdominal and thoracic surgeries by facilitating deep breathing and physiotherapy and preventing postoperative respiratory failure. The use of muscle relaxants is also one of the risk factors for postoperative pulmonary morbidity, and the use of RA can avoid it [21]. All these factors might have contributed to the early PACU discharge readiness in the STSA.

Most of the studies noted that hypotension was a concern, except for the study reported by Paliwal et al. [13]. The authors defined hypotension as a 30% fall from the baseline. Intraoperative hypotension is multifactorial, and the prevalence might be well affected by the variation of the definition used [22,23]. Our findings of high odds of hypotension with STSA might be explained by the involvement of cardio-acceleratory fibers affected by the techniques. Retrospective studies by Spannella F et al. and Vincenzi P et al. analyzing thoracic continuous spinal anesthesia in elderly patients of ASA-PS II-IV and administering as little as 2.5 mg bupivacaine or levobupivacaine (0.5 ml of 0.5% solutions) as bolus with adjuvants noted that more than 70% of the patients required norepinephrine infusion to maintain blood pressure [24,25]. As in most instances, spinal anesthesia leading to a heart rate (HR) fall is also associated with a fall in blood pressure (BP) and cardiac output (CO); such a fall may not be well tolerated by critical organs, especially in already cardio-respiratory compromised patients. A recent observational study from India analyzed data from 2,074 patients who underwent laparoscopic cholecystectomy under STSA. The study noted an incidence of 18% hypotension, 13% bradycardia, and 10% postoperative nausea vomiting [6]. However, these episodes were treated promptly and successfully with minimal effort. At the same time, it is unclear whether these short durations of low BP or low CO (dependent on HR, systemic vascular resistance, contractility, etc.) will significantly affect otherwise healthy patients. However, it might be harmful in at-risk patients with coronary artery disease or patients prone to cardiac demand-supply mismatch.

**Figure 5:**
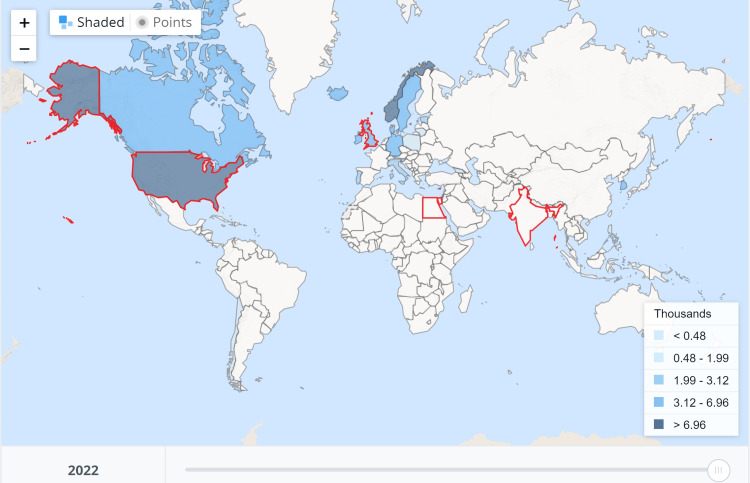
Per capita health expenditure per year, as mentioned in the World Health Organization Global Health Expenditure database for 2022. The World Health Organization retrieved the data on 15 April 2024; the image was taken on 21 August 2024. India and Egypt are among the countries that spend the least per capita (India approximately 50 and Egypt approximately 180 United States dollars). The countries marked with red boundaries are the United States of America, the United Kingdom, Egypt, and India. Image source: Worldbank (https://data.worldbank.org/indicator/SH.XPD.CHEX.PC.CD?view=map) (License: CC BY-4.0)

The present analysis, although it showed that STSA patients had intraoperative anxiety, shoulder pain, pruritus, and discomfort, the satisfaction reported was comparable to or better than GA. The interpretations, however, need to be judged against some aspects, as the assessments used the Likert or verbal rating scales. In contrast, perioperative patient satisfaction with the overall experience and anesthesia service is multifactorial, where factors beyond the health-related services also play a role and need to be assessed using questionnaires of multiple domains [26,27]. It has been found that even eye contact while a protocol or privacy is being breached leads to embarrassment and impacts emotional well-being [28]. Female patients often delay seeking healthcare for their breast-related diseases, including cancer. The survey indicates that female patients feel embarrassed even for radiotherapy, especially when a male therapist is present [29]. Sedation might help reduce intraoperative recall and avoid such embarrassing encounters, but it usually requires moderate to deep sedation [30]. STSA and deep sedation might take away many advantages of the technique over GA, as sedation and GA are a continuum, and moderate to deep sedation impacts cardio-respiratory functions [31]. However, healthcare delivery must consider multiple factors, including culture, care-providing setups, national economy, and cost. There were fewer procedure-related costs and hospital stays because of less postoperative pain and complications in the segmental thoracic spinal anesthesia [12]. Few other studies have also found that thoracic segmental spinal anesthesia is less expensive than GA [32,33]. Expert reviews and opinions suggest this unorthodox technique, i.e., STSA, is feasible, probably safe in selected patients, and economical for various abdominal and thoracic surgeries [5,34,35]. Our present analysis shows that the studies were primarily done in developing countries where per capita health expenditure is extremely low, as per the World Health Organization Global Health Expenditure database (Figure 5).

The present result, although derived from a small number of studies, has some practical implications. Patient selection and multidisciplinary discussion are crucial. The patients with higher risks of end-organ damage from hypotension, or where hypotension and bradycardia cannot be tolerated, need to be managed proactively and even might need prophylactic vasopressors to prevent such hypotension after STSA. Patients who are provided with the option of STSA as an alternative to GA will also require in-depth counseling regarding the surgical procedure performed while being awake and adequate sedation to make the patient comfortable. Surgeons co-operativeness is also equally crucial, especially while planning axillary dissection using cautery where excessive muscle twitching might be bothersome. 

Nonetheless, the STSA is trying to carve its space as an alternative to GA, and most studies conducted in the last few years also indicate a growing recent interest. Only the study by Gupta et al. [14] reported two cases requiring conversion to GA. The use of STSA for elective upper abdominal, thoracic, and breast surgeries in otherwise healthy patients, i.e., American Society of Anesthesiologists Physical Status (ASA-PS) I and II, can be a new thrust but requires sedation and hemodynamic support. Our study findings are, however, limited to a small number of eligible studies. Further, most of the studies included had some concerns with the methodology employed and had bias concerns. One of the studies might have been plagiarized, and the impact of the treatment might have been overestimated. Future studies should address the proper multidimensional assessment of patient satisfaction, and privacy concerns are warranted.

## Conclusions

The review and meta-analysis found very high odds of hypotension and bradycardia associated with STSA as compared to GA but significantly lower odds for PONV. STSA also demonstrated several other benefits, such as superior pain control, reduced opioid requirements, and shorter PACU stays. Further, it avoids the morbidity associated with tracheal intubation, which aligns with enhanced recovery after surgery (ERAS) principles. Issues such as intraoperative anxiety, shoulder pain, discomfort, and the need for sedation were noted, which could detract from patient comfort and overall satisfaction. The limited number of studies and methodological concerns, including risks of bias and lack of long-term outcome data, indicate that STSA should be cautiously approached, especially in sick patients or patients at risk of hypotension and bradycardia. Future research should focus on a more comprehensive assessment of STSA against well-conducted GA, including its impact on critical organ functions, long-term outcomes, and multidimensional patient satisfaction, to better understand its role in modern anesthetic practice. 
